# Infection and Transport of Herpes Simplex Virus Type 1 in Neurons: Role of the Cytoskeleton

**DOI:** 10.3390/v10020092

**Published:** 2018-02-23

**Authors:** Monica Miranda-Saksena, Christopher E. Denes, Russell J. Diefenbach, Anthony L. Cunningham

**Affiliations:** 1 Centre for Virus Research, The Westmead Institute for Medical Research, The University of Sydney, Westmead NSW 2145, Australia; cden1166@uni.sydney.edu.au; 2 Department of Biomedical Sciences, Faculty of Medicine and Health Sciences, Macquarie University, Sydney NSW 2109, Australia; russell.diefenbach@mq.edu.au

**Keywords:** herpes simplex virus, neurons, axonal transport, cytoskeleton, microtubules, actin

## Abstract

Herpes simplex virus type 1 (HSV-1) is a neuroinvasive human pathogen that has the ability to infect and replicate within epithelial cells and neurons and establish a life-long latent infection in sensory neurons. HSV-1 depends on the host cellular cytoskeleton for entry, replication, and exit. Therefore, HSV-1 has adapted mechanisms to promote its survival by exploiting the microtubule and actin cytoskeletons to direct its active transport, infection, and spread between neurons and epithelial cells during primary and recurrent infections. This review will focus on the currently known mechanisms utilized by HSV-1 to harness the neuronal cytoskeleton, molecular motors, and the secretory and exocytic pathways for efficient virus entry, axonal transport, replication, assembly, and exit from the distinct functional compartments (cell body and axon) of the highly polarized sensory neurons.

## 1. Introduction

Viruses are acellular, obligate intracellular parasites that can only reproduce within living hosts, and hence have evolved to utilize different mechanisms of viral reproduction. All the viruses have a capsid, which contains either double-stranded or single-stranded DNA or RNA. The shape of the capsid itself varies between viruses, and capsids are either enclosed within an envelope or naked (lacking an envelope). In order to reproduce, the virus must enter the host cell. Once inside the cell, the virus may use the host cell cytoskeleton, that is the actin and microtubule networks, for navigation through the cell cytoplasm [1,2]. The viral life cycle begins with the attachment of the virus to the host cell membrane, which allows for the virus or viral particles to enter the cell. The viral genome then needs to be translocated to the nucleoplasm or cytoplasm where replication of the viral genome, and the synthesis of viral proteins, takes place. These then move to sites where assembly of viral particles occurs, and finally the virus is transported back to the cell plasma membrane. This process usually involves the virus using the cell’s secretory pathways and the cytoskeleton, which are important for long distance transport of cellular cargo and organelles [1,3].

## 2. Herpes Simplex Viruses

The herpes viruses are DNA viruses able to cause lytic and latent infections in both human and animal populations. All of the herpesviruses (more than 100 known viruses) share a common virion morphology and a group of conserved genes that are required for virus replication [4]. Herpes simplex virus type 1 and 2 (HSV-1 and HSV-2) are human neuroinvasive pathogens and are amongst the most commonly studied members of the alphaherpesviruses. The spread of alphaherpesviruses through the peripheral nervous system is critical for establishing a life-long infection within sensory neurons of the host. HSV-1 is approximately 225 nm in diameter and consists of four layers: the episomal DNA genome, capsid, tegument and envelope [5]. At the center is a large linear double-stranded DNA (dsDNA) genome (152 kbp) that codes for more than 80 open reading frames (ORFs) enclosed within a T = 16 icosahedral capsid. Surrounding the capsid is a dense proteinaceous layer called the tegument consisting of 23 tegument proteins that link the capsid to the host cell-derived lipid-bilayer envelope containing 16 different membrane proteins [6,7,8].

**Figure 1:**
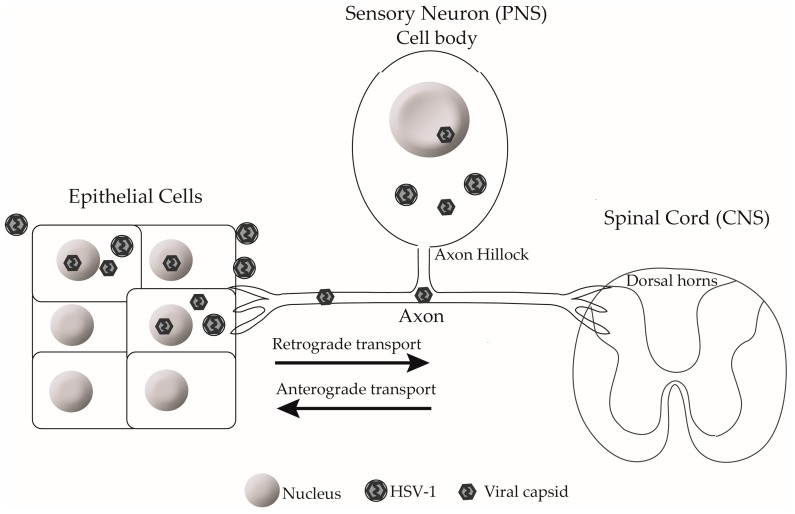
Herpes simplex virus type 1 (HSV-1) life cycle in the human host. After primary infection in the epidermis, HSV-1 enters sensory nerves innervating the skin or mucosa and undergoes retrograde axonal transport to the neuronal cell body where it establishes a life-long latent infection within sensory neurons. During reactivation, HSV-1 travels via anterograde axonal transport towards the peripheral epidermis to cause recurrent herpes or to be asymptomatically shed.

Primary HSV-1 infection is usually established within the stratified squamous epithelium (epidermis) of the oral and anogenital mucosa and is commonly asymptomatic (Figure 1). Upon infection of sensory nerves innervating the skin or mucosa, the virus undergoes retrograde axonal transport to the neuronal cell body where it can establish a life-long latent infection within the trigeminal and dorsal root ganglia (DRG) [9,10]. After reactivation, anterograde transport of HSV-1 is usually directed towards the peripheral epithelial cells where it typically presents as mild oral or labial lesions in an immunocompetent host (Figure 1) [11]. In neonates or immunocompromised individuals, however, lesions are more severe and persistent, often causing serious and life-threating diseases, such as herpes simplex encephalitis and disseminated herpes. Even in immunocompetent individuals, HSV-1 keratitis, which is an infection of the cornea in the eye, can result in blindness [12]. Prior genital infection with HSV-2 also enhances HIV acquisition 3–5 fold [13].

## 3. Dorsal Root Ganglia Neurons

Sensory DRG neurons have pseudounipolar axons that bifurcate outside the DRG to innervate the spinal cord and the epithelium (Figure 1). DRG neurons are encapsulated by a glial cell sheath, which is formed from closely applied satellite cells, which are in turn encased in a basal lamina [14]. The neuron-satellite cell unit is separated from other similar units by collagen fibrils and other extracellular matrix molecules. The structure of a DRG neuron consists of a cell body connected to the axon by the axon hillock (Figure 1). The cell body contains the nucleus, which is where viral DNA replication, transcription, and capsid assembly takes place during HSV-1 infection (Figure 1). The axon serves to conduct the transmission of action potentials from the cell body to synapse. It also provides a physical conduit for the transport of essential biological materials from the cell body to the synapse, which is necessary for the function and viability of neurons. The axon hillock is a specialized neck region that contains key organelles and a specialized dense cytoskeletal organization, which controls the axonal transport of proteins and mRNA. The axon hillock also forms a barrier, where organelles such as Golgi bodies and lysosomes accumulate in the cell body, being excluded from the axoplasm. Other organelles, such as synaptic vesicles and mitochondria, are able to freely pass through the barrier and be transported into axons [15,16].

Long distance transport in axons is primarily dependent on microtubules. The motor proteins that mediate axonal transport on microtubules are members of the kinesin and dynein superfamilies [17]. Kinesins are generally plus-end directed motor proteins that transport cargoes such as synaptic vesicle precursors and membranous organelles in anterograde direction towards the synapse. Cytoplasmic dyneins are minus-end-directed motors that transport cargoes like neurotrophic signals, endosomes, and other vesicles retrogradely towards the cell body. Based on kinetics of transport, axonal transport is classified as either fast or slow [18,19]. Fast axonal transport occurs at a rate of 0.5–10 µm/s in both anterograde and retrograde directions and includes the transport of mitochondria, neurotransmitters, membrane bound organelles, channel proteins, multi-vesicular bodies, and endosomes. In contrast, slow axonal transport occurs in the anterograde direction at a rate of 0.01–0.001 µm/s and transports cytoskeletal components, like neurofilaments, tubulin and actin, as well as proteins such as clathrin [18,20].

Maintenance of the highly polarized nature of neurons into distinct functional domains (cell body and axons) involves the coordinated regulation of the cytoskeleton (mainly microtubules and actin) and membrane trafficking machinery [21]. In addition to herpesviruses, such as varicella-zoster, HSV-1 and pseudorabies (PRV), many other viruses like rabies and West Nile viruses have evolved the ability to undergo long-distance directed and coordinated travel along axons in both retrograde and anterograde directions by utilizing the cytoskeleton and the neuronal trafficking machinery for efficient infection of the peripheral nervous system [22,23,24,25,26,27].

## 4. HSV-1 Entry into Neurons

**Figure 2:**
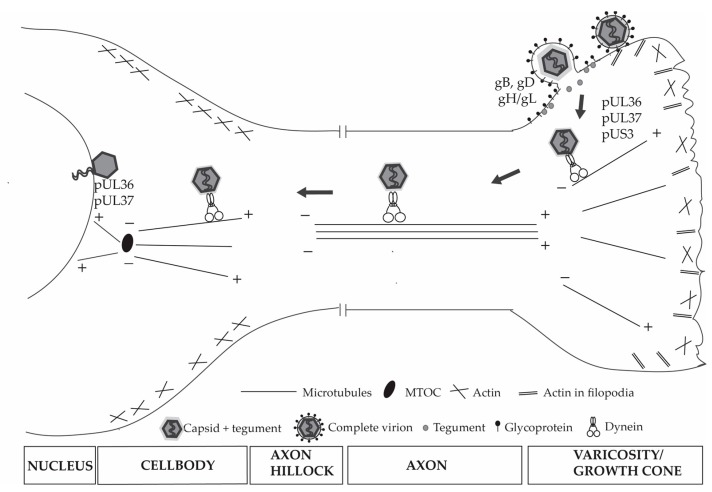
Entry of HSV-1 into neurons. HSV-1 enters neuronal cells by attachment and fusion of the viral envelope with the plasma membrane and this process is mediated by viral glycoproteins gB, gD, and gH/gL. During membrane fusion, the HSV-1 envelope and its glycoproteins remain associated with the plasma membrane while most tegument proteins dissociate from the incoming capsid. The capsid with associated inner tegument pUL36, pUL37, and pUS3 traverses the actin cortex and is transported along microtubules towards the MTOC to the nucleus in the cell body. The retrograde transport of incoming capsids to the nucleus is mediated by the cellular motor dynein and requires pUL36 and pUL37. MTOC, microtubule organizing center.

The mechanism of HSV-1 entry into cells differs depending on the cell type and is mediated by the direct interaction between viral envelope glycoproteins and cell surface receptors. HSV-1 enters human epidermal keratinocytes by either pH-dependent endocytosis or by a nectin-1 receptor- dependent membrane fusion pathway under low temperature [28], while entry into neurons is mediated by pH-independent fusion of the HSV-1 envelope with the neuronal plasma membrane (Figure 2) [29,30,31]. The initial attachment of the virus to the cell plasma membrane is mediated by the binding of the viral glycoproteins B and C (gB and gC) to cell surface “concentrating receptor” heparan sulfate proteoglycans. Viral glycoprotein D is the major viral attachment protein binding to the herpesvirus entry mediator (HVEM), a member of the tumor necrosis factor (TNF) receptor family, 3-O-sulfated heparan sulfate and nectin-1 (also known as HveC and CD111), a cell adhesion molecule in the immunoglobulin superfamily. The interaction of these viral glycoproteins with cell receptors triggers the fusion of the viral envelope with the cell plasma membrane via the activator of cell entry, the heterodimeric gH/gL, and the fusion receptor gB [30,31,32,33,34,35,36,37,38].

Regardless of the mechanism of entry, the incoming capsid first encounters the host cell actin cytoskeleton lining the cytosolic side of the plasma membrane containing a meshwork of filamentous actin (F-actin) connected to the plasma membrane through surface receptors, like nectins and heparan sulfate. Cryo-electron tomography studies showed the presence of incoming HSV-1 capsids surrounded by a meshwork of F-actin filaments in synaptosomes (severed axon termini) [39]. This actin meshwork provides a physical barrier to the entry of viruses into host cells, and hence, viruses like HSV-1 have evolved mechanisms to alter and utilize the actin cytoskeleton to facilitate their entry and replication in host cells.

### 4.1. Viral Surfing along Filopodia

The initial site of entry within neurons is usually at the axon terminus near peripheral epithelial cells (Figure 1), but in vitro, entry can also occur at the cell body [40]. HSV-1, like the related swine pseudorabies virus (PRV) and other unrelated non-neurotropic viruses, such as HIV, exploits the cytoskeleton to travel long distances from the site of entry towards cell body. HSV-1 utilizes the actin rich swellings or varicosities along axons as well as the extensive network of thin actin rich membrane protrusions known as filopodia in both neuronal and non-neuronal cells for efficient viral travel or ‘surfing’ to reach the cell body [40,41,42,43,44]. Filopodia are involved in various cellular processes, such as cell migration, wound healing, neurite outgrowth, and guidance. Filopodia are formed by stiff, tightly bound parallel actin bundles, which polymerize at the barbed end of filaments towards the plasma membrane [45,46,47,48]. Filopodia are highly motile frequently extending and retracting and this process is controlled by a dynamic balance between the ongoing actin polymerization at the end of the filaments and the retrograde flow of F-actin bundles [49].

HSV-1 envelope protein gB attachment to heparan sulfate, which is abundant in filopodia, has been shown to be required for viral binding to filopodia and viral surfing in non-neuronal cells. Exposure to HSV-1 induces the formation of filopodia in non-neuronal cells and PRV induces the formation of varicosities in neuronal cells [41,43,50]. The induction of filopodia formation during HSV-1 infection is regulated by the transient activation of the small Rho GTPase Cdc42. Rho GTPases are key regulators that link cell surface receptors with the actin cytoskeleton [51,52,53]. RhoA, Rac1, and Cdc42 are Rho GTPases, which have been described to be involved in HSV-1 entry into neuronal and non-neuronal cells [43,50,54,55]. Activation of Rho A leads to the formation of actin stress fibers, while activation of Rac1 and Cdc42 induces the formation of lamellipodia and filopodia, respectively [51,52]. HSV-1 entry into neurons is dependent on gD binding nectin-1. Binding of PRV gD to nectin-1 has been shown to induce the activation of Rho GTPase Cdc42, which in turn, triggers the reorganization of the actin cytoskeleton and induces the formation of axonal varicosities that are sites of HSV-1 and PRV entry and egress along axons [40,43,56,57,58]. In addition, long distance axonal transport of PRV requires rapid local protein synthesis of cellular trafficking proteins including the vesicle transport regulator Anxa2, intermediate filament component Prph, and the dynein regulator LIS1 [59]. Many viruses, including Epstein-Barr, human herpesvirus-8, HIV-1, influenza, respiratory syncytial, and dengue (type 2) viruses have also been reported to activate Cdc42 for reorganization of the actin cytoskeleton to facilitate virus infection and spread between host cells [60,61,62,63,64,65,66].

### 4.2. HSV-1 Exploits the Neuronal Cytoskeleton and Motor Proteins

Upon entry into neuronal cells, HSV-1 has also been described to hijack cofilin-1, a member of the actin depolymerizing factor ADF/cofilin family, which is primarily responsible for actin reorganization. During infection of neurons, HSV-1 causes a biphasic remodeling of the actin cytoskeleton by causing the inactivation of cofilin-1 followed by its subsequent activation, resulting in F-actin assembly and disassembly during early and late stages of infection, respectively [60,67]. The binding of HSV-1 to the host cell membrane triggers the phosphorylation of cofilin-1 by the epidermal growth factor receptor-phosphatidylinositide 3-kinase (EGFR-PI3K) and downstream mediators, the extracellular signal-regulated kinase (ERK), Rho-associated coiled-coil-containing protein kinase 1 (ROCK), and LIM kinases (Erk1/2-ROCK-LIMK) signaling pathways, which inactivates cofilin-1 and induces F-actin polymerization to facilitate virus entry. Subsequent viral penetration induces the activation of cofilin-1, leading to the fragmentation of existing actin filaments to facilitate virus trafficking [60,67].

The dynamics of F-actin transport is regulated by myosins that are mechanoenzymes that hydrolyze ATP to generate movement of actin filaments and transport of cellular components along the actin cytoskeleton [68]. Myosins form a large family and are grouped into classes based on their motor domains. Transport along filopodia during virus surfing is dependent on the underlying actin cytoskeleton and is regulated by myosin II, which is a plus end motor involved in the regulation of F-actin dependent-movement towards the cell body (F-actin retrograde flow) [68,69]. The heavy chain subunits of the non-muscle myosin IIA and IIB (NMHC-IIA and NMHC-IIB) have been shown to be co-receptors for HSV-1 by interacting with gB to mediate membrane fusion and virus entry into non-neuronal cells [70,71]. HSV-1 entry appears to upregulate the surface expression of NMHC-IIA and NMHC-IIB, but the mechanism of this upregulation is unknown. It is believed that HSV-1 entry triggers the release of Ca^2+^ and results in the activation of myosin light chain kinase (MLCK), which in turns controls the surface expression of NMHC-IIA and NMHC-IIB by phosphorylation [70,71]. Myosin IIB is highly expressed in neurons, and it therefore remains to be investigated whether it also may play a role in HSV-1 entry into neurons [72].

Upon membrane fusion and release of the capsid into the cytoplasm, the HSV-1 envelope, and its glycoproteins remain associated with the plasma membrane and most tegument proteins dissociate from the capsid upon its release into the cytoplasm of the cell (Figure 2) [40,73,74,75]. The capsid is transported via retrograde transport from the cell periphery along microtubules towards the nucleus and this transport appears to be mediated by the molecular motor dynein/dynactin complex. Initiation of retrograde transport of HSV-1 in human non-neuronal cells has been described recently to utilize a dynamic microtubule plus end complex comprising the end binding protein EB1, cytoplasmic linker protein 170 (CLIP-170), and dynactin-1 (DCTN1). These plus end tracking proteins (known as +TIPS) control microtubule dynamics and stability at the plus end of microtubules and play a role in the interactions of microtubules with organelles and cargo [76]. The mechanisms by which viruses, like HSV-1, engage microtubules and molecular motors like dynein/dynactin for retrograde transport in neurons remains unclear and whether HSV-1 exploits the +TIPS complex remains to be elucidated.

Various viral proteins have been described to interact with dynein in vitro, including HSV-1 pUL9, pUL34, and pUL35. HSV-1 pUL9 was found to bind to the cytoplasmic dynein 1 light chain LC8, while pUL34 interacts with dynein intermediate chain (DYNC1I) 1a [77,78]. The interactions of pUL9 and pUL34 with dynein are unlikely to play a role in the retrograde transport of HSV-1 as they are not part of the mature virions. The HSV-1 capsid protein pUL35 was found to interact with the dynein 1 subunits, Tctex1 and RP3 in vitro [79]. However, studies have shown that dynein mediated retrograde transport of both HSV-1 and PRV capsids occurs in vitro efficiently in the absence of pUL35 and most tegument proteins [80,81]. Dynein, dynactin, and kinesin-1 have been shown to bind directly, simultaneously, and independently of other host proteins to HSV-1 capsids purified from extracellular virions containing exposed inner tegument proteins, including pUS3, pUL36, pUL37, ICP0, pUL14, pUL16, and pUL21, and this binding promoted capsid movement along microtubules independently of dynactin [82,83]. Purified capsids from the nucleus were shown not to bind to dynein or kinesin-1, but were able to bind directly to dynactin although this interaction with dynactin did not enhance capsid recruitment of dynein. Hence, these findings indicated the involvement of inner tegument proteins in the recruitment of molecular motor for the transport of virus capsids along microtubules [82,83].

The viral inner tegument proteins pUL36, pUL37, and pUS3 have been shown to be co-transported with HSV-1 and PRV capsids along microtubules. pUL36 and pUL37 are important for promoting neuroinvasion by guiding the viral capsids along microtubules to the nucleus and are required for virion assembly during egress [84,85,86,87,88,89,90]. Therefore, pUL36 and pUL37 have been considered to be the clear effectors for virus interaction with molecular motors, such as dynein and kinesin. pUL36 recruits and tethers the capsid to the dynein/dynactin motor complex after virus entry. A proline-rich region in the C-terminal half of PRV pU36 has been shown to directly interact with dynein/dynactin complex and to be sufficient for the retrograde axonal transport of PRV capsids (both in vitro and in vivo) [84]. In addition to pUL36, pUL37 has been recently described to direct sustained capsid axonal retrograde transport along microtubules to the neuronal nucleus, which is consistent with its requirement for rapid nuclear translocation of incoming capsids in non-neuronal cells [84,85,91]. One of three conserved surface-exposed regions of pUL37, the R2 region was reported to rectify the bidirectional movement of incoming HSV-1 and PRV capsids (due to their engagement of both dynein and kinesin motors) along axons to a retrograde-biased direction towards the nucleus [85].

Interestingly, pUS3, pUL36, and pUL37 proteins, which remain associated with the virus during entry, also play key roles in HSV-1 immune evasion. The viral protein kinase pUS3 is an inhibitor of the NF-κB pathway and also of the toll-like receptor 2 (TLR2) signalling pathway [92,93]. pUL36 has a ubiquitin-specific protease (UL36USP) activity that has been shown to inhibit beta interferon production by deubiquitinating the TNF receptor associated factor 3 (TRAF3) [94]. pUL36 has also been reported to abrogate the DNA sensing signalling pathway by inhibiting NF-κB activation to evade host antiviral innate immunity [95]. pUL37 is a viral protein deamidase which inactivates the retinoic acid inducible gene I (RIG-I) signalling pathway and prevents it from sensing viral dsRNA and subsequent antiviral cytokine production [96].

## 5. Viral Capsid Assembly, Transport and Egress from Nucleus

Once the capsid reaches the nucleus, pUL36 plays a role in capsid docking onto nuclear pores, as well as the release of the dsDNA genome inside the nucleus [84,97,98,99,100]. Viral DNA is released into the nucleus through the nuclear pores and the linear genome is circularized to serve as a template for new DNA synthesis. Viral replication, late gene expression, and capsid formation take place within distinct intranuclear structures, called replication compartments. A recent study reported that nuclear import of the infected cell protein 4 (ICP4) (important for viral early and late transcription), capsid assembly in the nucleus and egress into the cytoplasm of neurons (and also in non-neuronal cells) require importin α1, which is a member of the importin family of proteins involved in the nuclear protein import [101]. Importin α1 also plays a role in the nuclear import of various other HSV-1 proteins in non-neuronal cells, including ICP0 (required for viral transcription), ICP8, pUL30, pUL42 (three HSV-1 proteins important for viral replication), and pUL31 (required for virus budding at the inner nuclear membrane) [101,102,103].

Nucleus expansion, lamina disruption, and chromatin dispersal occur alongside viral replication compartment formation [104]. The mechanisms controlling the formation of these compartments and the nucleus expansion, are, however, poorly understood. G-actin has been shown to be essential for nuclear expansion and disruption of the chromatin during HSV-1 infection of non-neuronal cells, while the expression of the viral proteins pUL31 and pUL34 is required for lamina disruption [104,105]. These changes in the nucleus induced by HSV-1 are believed to facilitate movement of the capsids to the nuclear membrane for nuclear egress. Movement of capsids in the nucleus is active and directed as well as energy- and temperature-dependent [106]; however, the mechanism of capsid intranuclear movement remains unclear, specially whether or not it is actin-dependent. Studies have shown that movement of GFP labelled capsids in HEp-2 cells was F-actin-dependent and that PRV and HSV-1 infection of neurons induced the formation of actin filaments in the nucleus, which associated with capsids [106,107]. However, a recent study showed that nuclear capsid movement is independent of F-actin [108]. These contradictory results were attributed to the use of actin inhibitor latrunculin A (LatA), which causes the formation of actin rods in some cells but not in others. These actin rods were found to bind to capsids and inhibit their motility, and therefore capsid motility is blocked by LatA only if actin rods are formed like in fibroblasts. In addition, treatment with cytochalasin D, an inhibitor of actin polymerization, reversed the formation of actin rods and did not significantly inhibit the intranuclear movement of capsids. These findings suggest that intranuclear capsid movement is independent of F-actin [108]. No molecular motors have yet been shown to direct movement of intranuclear capsids despite evidence showing capsid co-localization with Myosin V in the nucleus. Furthermore, treatment with the putative myosin ATPase inhibitor 2.3 butanedione monoxime (BDM) results in a decrease in the rate of capsid transport. However, these studies have been inconclusive due to questions in the specificity of BDM for myosin ATPase [106,107]. Hence, further studies are required to elucidate the mechanisms of how capsids move within the nucleus and the role of the nuclear actin, actin rods, and myosins in capsid transport to the nuclear membrane for exit. These studies are complicated by the difficulty of imaging nuclear actin and the current debate on the nature and role of actin in the nucleus [109,110].

The fully assembled capsid exits the nucleus by budding through the inner nuclear membrane and fusing with the outer nuclear membrane. Primary envelopment occurs at the inner nuclear membrane, and de-envelopment occurs via fusion of the primary envelope with the outer nuclear membrane to release the naked capsid into the cytoplasm. This process is mediated by the nuclear egress complex (NEC) consisting of pUL31 and pUL34 and their interaction with other viral and cellular proteins, including viral proteins γ134.5, pUS3, and pUL47, and cellular p32 and protein kinase C [111,112,113]. pUL31 binds to pUL34, a type II transmembrane protein, which is then targeted to the nuclear envelope thereby linking the capsid to the inner nuclear membrane [114,115]. The complex then recruits pUS3, a viral protein kinase, which along with the cellular protein kinase C, phosphorylates nuclear lamins resulting in the dissolution of the nuclear lamina at the budding sites [116,117].

## 6. HSV-1 Assembly in the Cytoplasm of the Cell Body

**Figure 3:**
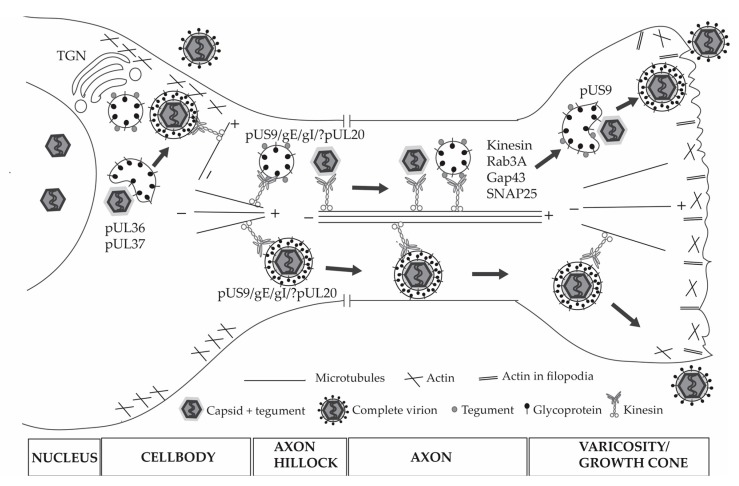
HSV-1 assembly, transport and exit from neurons. In the cell body, following nuclear egress, naked capsids acquire inner tegument proteins predominantly in the cytoplasm followed by the acquisition of outer tegument and envelope proteins as capsids invaginate vesicles derived from the TGN. The fully enveloped virion is transported to the plasma membrane where it can exit the cell body. Viral capsids containing inner tegument proteins with or without an envelope and vesicles associated with glycoproteins and tegument proteins are also targeted for active transport along microtubules in axons utilizing the neuronal secretory pathway (involving Rab3A, Gap43, SNAP25, and kinesin) and this process is mediated by the envelope proteins pUS9, gE/gI and possibly (?) pUL20. Viral capsids acquire a final envelope by invagination of vesicles with associated tegument and envelope proteins prior to exit from axonal growth cones.

Following nuclear egress, naked capsids acquire tegument proteins predominantly in the cytoplasm of the cell. There are studies indicating a partial localization and addition of some tegument proteins to capsids in the nucleus, but these observations have not been supported by other studies [118,119,120,121,122]. It is, however, generally agreed that during capsid maturation in the cytoplasm, tegument addition is at least a two-step process. Inner tegument proteins bind first to the capsid, while outer tegument proteins are added during secondary envelopment when the capsids invaginate vesicles derived from the *trans*-Golgi network (TGN) containing envelope glycoproteins [122,123]. This gives rise to a fully enveloped virus contained within a transport vesicle (Figure 3). Binding of the inner tegument proteins pUL36 and pUL37 to capsids is essential for secondary envelopment and directed movement of the capsids along microtubules in both non-neuronal and neuronal cells [86,87,88,89,90,124,125,126]. Deletion of either pUL36, pUL37, or their binding regions blocks secondary envelopment resulting in an accumulation of naked capsids in the cytosol which fail to proceed to sites of secondary envelopment. These findings suggest that capsids failed to engage molecular motors for transport along microtubules and indicate a role for pUL36 and/or pUL37 in the recruitment of kinesins during virus assembly and egress.

pUL36 has been shown to play a role in the recruitment of kinesin to both naked capsids with exposed inner tegument proteins and TGN-derived membrane associated capsids and to promote their transport along microtubules using in vitro systems [82,124,127]. Although pUL37 has not yet been found to directly interact with kinesin, it has been shown to interact with dystonin/BPAG1, in a yeast two-hybrid system [128]. Dystonin is a large protein belonging to the conserved spectraplakin family of proteins that are involved in the anterograde transport of vesicles, stabilization of microtubules via its interaction with the microtubule associated protein 1 (MAP1), and the retrograde transport in neurons via its interaction with the p150^glued^ subunit of dynactin [129,130,131]. Dystonin depletion resulted in a block of capsid transport in the cytoplasm resulting in the accumulation of capsids and a delay in virus production [128]. The phenotype that was caused by depletion of dystonin was similar to that when microtubules are disrupted by treatment with nocodazole with virus production eventually reaching normal levels, suggesting that HSV-1 uses alternative mechanisms to exit from cells when microtubules are disrupted [128]. There are various isoforms of neuronal specific dystonin, and whether pUL37 interacts with dystonin in neurons and whether this interaction plays a role in HSV-1 axonal transport remains to be determined [132].

Other viral proteins have been described to interact with kinesin. For example, HSV-1 tegument protein pUS11 interacts with kinesin-1 heavy chain (KIF5B) and KLC-like protein PAT1 while HSV-2 membrane-associated tegument protein pUL56 binds the kinesin-3 member KIF1A; however, both of the proteins are dispensable for virus propagation [133,134,135]. Recently, pUS3 was shown to phosphorylate kinesin-2 (KIF3A) to downregulate the CD1d cell surface expression in HSV-1 infected cells by suppressing its recycling which is dependent on kinesins [136]. CD1d is a key antigen presenting molecule required for natural killer T cell (NKT) activation, and therefore, downregulation of CD1d is key for HSV-1 evasion of NKT and serves to protect infected neurons in the DRG from destruction [137,138]. In addition, pUS3 also phosphorylates the HSV-1 tegument protein pUL49, which has been shown to be important for virus spread in non-neuronal cells in vitro and in vivo in mouse cornea [139,140]. pUL49 induces the stabilization and hyperacetylation of microtubules to promote microtubule associated trafficking of proteins by promoting kinesin-1 binding and transport [141,142].

Viral envelope acquisition is generally considered to occur at the TGN. The process by which envelope glycoproteins are transported to the sites of envelopment is not yet fully defined. Some (i.e., HSV-1 gB and gE) but not all envelope proteins contain trafficking motifs which are predicted to direct their cellular localization [143,144,145]. Those envelope proteins without trafficking motifs have been described to require other viral proteins for their transport and incorporation into capsids at the sites of envelopment. For example, gD and gH/gL require gM and gK/pUL20 complex for their localization at assembly sites in the cytoplasm [146]. Several cellular vesicular transport pathways have been described to be utilized by HSV-1 for transport of envelope proteins to the sites of envelopment in non-neuronal cells, including the endosomal sorting complex required for transport (ESCRT) and dynamin-dependent endocytosis [147,148,149,150]. Although the involvement of these pathways for the transport of HSV-1 envelope proteins to envelopment sites remains to be confirmed in neurons, studies have shown that tegument and envelope proteins of both HSV-1 and PRV traffic with vesicles containing rab6, rab3A, rab8, rab11a, GAP-43, SNAP-25, which are proteins of the secretory vesicular pathway in the neuronal cell body and axons [56,151,152].

After final envelopment, the enveloped capsid is transported along microtubules towards the plasma membrane where the capsids must navigate the cortical actin cytoskeleton to get to the plasma membrane. Myosins, including myosin Va and non-muscle myosin II, have been implicated in the transport of enveloped capsids within vesicles and vesicles bearing viral glycoproteins from the TGN to the plasma membrane and to play a role in virus egress and cell surface expression of viral glycoproteins from non-neuronal cells [153,154]. It is expected that myosins will also be involved in the trafficking of HSV-1 to the plasma membrane in neuronal cells as the actin cytoskeleton is modified by HSV-1 via cofilin-1 to facilitate its infection of neurons [60].

## 7. HSV-1 Anterograde Transport and Exit from Axons

In neurons cultured in vitro, the virus can exit the neuron either at the cell body or the axons. However, in vivo, HSV-1 is targeted for active transport along axons to the nerve endings for virus spread to other hosts after reactivation from latency. The mechanism involved for axonal sorting, targeting and transport of HSV-1 is debated with some clear differences to that of PRV.

For HSV-1, viral particles have been shown along axons either as naked capsids without any membrane (Subassembly or Separate model) or as fully enveloped capsids within vesicles (Married model), while PRV has been shown to be transported along axons mainly as fully enveloped capsids (Married model) (Figure 3) [24,56,57,155,156,157,158,159,160]. Efficient targeting of PRV viral particles and some glycoproteins (gB, gC, gD and gE) to axons requires the envelope protein pUS9, which contains sorting motifs in the acidic domain in its cytoplasmic tail. The association of PRV pUS9 with lipid rafts is required for transport of PRV structural proteins and for PRV spread from pre-synaptic to post-synaptic neurons [161,162,163,164,165]. In the case of HSV-1, pUS9 plays more than one role in HSV-1 infection of neurons: it is important for efficient anterograde axonal transport of HSV-1 capsids and also plays a role in virus assembly in varicosities and growth cones of human axons [160,166]. However, pUS9 does not act alone, but in conjunction with gE/gI to promote the sorting of and long distance axonal transport of HSV-1 capsids and glycoproteins [167,168]. A recent study also suggests the possible involvement of the HSV-1 membrane protein pUL20 in targeting HSV-1 capsids (and not glycoproteins or at least gD) to axons in adult mice DRG neurons as deletion of pUL20 resulted in fewer HSV-1 capsids entering axons. This study also reported that inner tegument proteins pUL36 and pUL37 are sufficient for the efficient capsid transport in the cytoplasm of the cell body, but not for capsid targeting to axons [169]. Overall, further studies are required to fully understand the mechanisms involved in HSV-1 transport along and exit from neuronal axons and the contribution(s) of viral and cellular proteins.

Regardless of the models proposed, HSV-1 utilizes the axonal cytoskeleton and engages molecular motors, like kinesins, for active and directed transport for spread from neurons. Viral capsids and vesicles bearing viral glycoproteins are observed in close association with microtubules and their transport along axons is microtubule dependent [57,127,149,170]. Studies using time-lapse microscopy have shown that axonal transport of HSV-1 and PRV occurs by fast axonal flow (approximately 2 μm/s for PRV) and to be bidirectional [171,172]. Using immuno-electron microscopy, label for kinesin-1 has been seen associated with capsids and vesicles bearing tegument and envelope proteins along axons and in varicosities and growth cones [56]. The basic domain of HSV-1 pUS9 has been shown to directly bind kinesin-1 and this binding is important for HSV-1 anterograde axonal transport and virus egress from neuronal growth cones and varicosities [173]. PRV pUS9 has been shown to bind kinesin-3 (KIF1A), and this binding requires the presence of glycoproteins gE and gI [174,175]. HSV-1 pUS9 has not been shown to directly bind kinesin-3 and when introduced into a US9 deletion mutant PRV, it failed to bind to kinesin-3, suggesting fundamental differences in HSV-1 and PRV pUS9 [175].

The molecular mechanisms involved in the exocytosis of viral particles from the cell body and along axons in neurons remains to be fully elucidated. Virus egress from cells involves the fusion of the transport vesicle membrane containing the fully assembled virus with the host plasma membrane. Using total internal reflection fluorescence microscopy (TIRF) and pH-sensitive probes to label viral proteins, PRV viral particles, vesicles with associated glycoproteins, and L-particles used the same constitutive secretory pathways associated with Rab6a, Rab8A, and Rab11a to exit non-neuronal and unpolarized cells [176]. In DRG neurons, HSV-1 viral particles, and vesicles bearing tegument and envelope proteins associate with key proteins involved in the large secretory and exocytic pathway including Rab3A, GAP-43, and SNAP-25 along axons to reach and exit from growth cones and varicosities [56]. These proteins play important roles in intracellular trafficking, docking and fusion of vesicles to the plasma membrane and regulation of exocytosis [177,178,179]. In addition, kinesin-1 was found associated with these vesicles transporting viral tegument and envelope proteins and viral particles along axons and within vesicles inside growth cones and varicosities. Furthermore, Rab3A, GAP-43, and SNAP-25 were also found incorporated into the membrane of the extracellular virions. In addition, vesicles that were associated with the trafficking of PRV pUS9 were found to have the vesicle and target soluble NSF attachment protein receptors (SNAREs) including vesicle-associated membrane protein 2 (VAMP2), synaptosomal-associated protein 25 (SNAP-25), vesicle transport through interaction with t-SNAREs homolog 1B (VTI1B), and Syntaxin-6 incorporated into their membrane [175]. Together, the above findings provide evidence supporting the concept that fully enveloped viral capsids in growth cones and varicosities utilize the large vesicle pathway of exocytosis to exit the axons.

## 8. Concluding Remarks

Overall, the research findings discussed in this review give a glimpse of how HSV-1 (and also PRV) has evolved mechanisms to subvert the host cell cytoskeleton and secretory pathways for their efficient transport along sensory axons to deliver the viral genome to the nucleus and subsequent spread from the nucleus along axons to epithelial cells after reactivation. HSV-1 utilizes actin and microtubules for both retrograde transports from the plasma membrane and along axons during virus entry and for anterograde transport during virus assembly and exit. The composition of the viral particles, especially their protein complement, appears to determine which molecular motors the virus engages and the direction of transport during entry and exit of the virus in neuronal and non-neuronal cells.

The unique neuroinvasive properties of HSV-1 have allowed its development as gene delivery vector for the treatment of disorders and diseases of the nervous system. These properties include the episomal non-integrating DNA genome containing several dispensable genes and the ability to established life-long latent infections in sensory neurons. Further research is needed to unravel how HSV-1 exploits and interacts with the neuronal cytoskeleton components and the vesicular secretory and exocytic pathways in these highly polarized human cells. Understanding these viral mechanisms will contribute to the further development of HSV gene therapy vectors and new antiviral strategies for the treatment of recurrent herpes infections and to the elucidation of the pathogenesis of other neurotropic viruses, including other herpesviruses (i.e., varicella-zoster, Epstein-Barr, and cytomegalovirus) and non-herpesviruses, like rabies and poliovirus, whose infection also depends on harnessing the host cellular cytoskeleton.
